# Supplementary material for: Two-tier supramolecular encapsulation of small molecules in a protein cage
Source: Nat Commun. 2020 Oct 26;11:5410. doi: 10.1038/s41467-020-19112-1 (PMC7588467; doi:10.1038/s41467-020-19112-1)
Supplement: Supplementary file 1 — Supplementary Information [file 41467_2020_19112_MOESM1_ESM.pdf]

Supplementary Information

Two-tier supramolecular encapsulation of small molecules in a protein cage

Thomas G.W. Edwardson, Stephan Tetter, Donald Hilvert*

*Correspondence to: donald.hilvert@org.chem.ethz.ch

**Contents**	**Pages**
Supplementary Figures 1-15	S2 – S15
Supplementary Table 1	S16

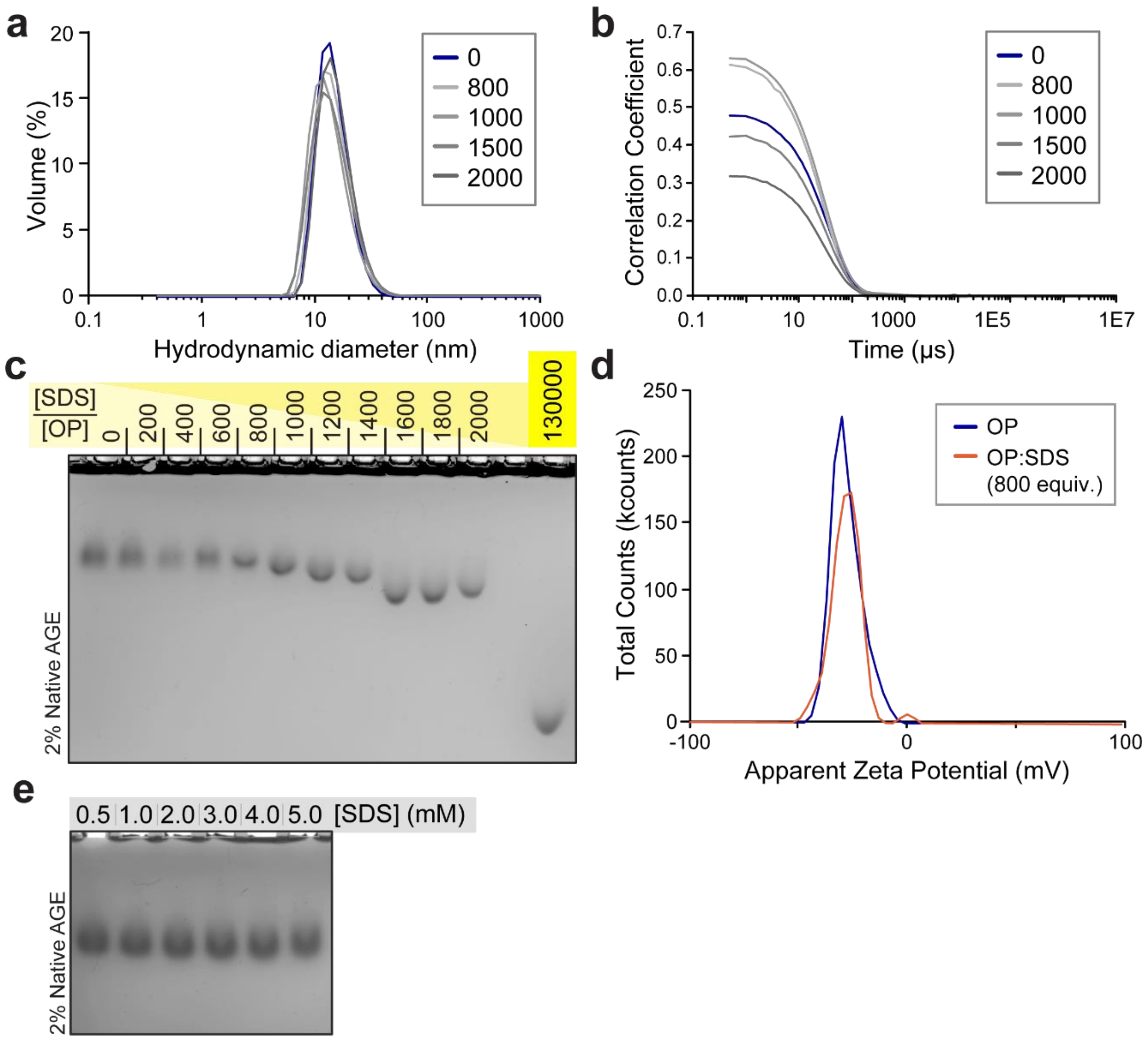

Supplementary Figure 1. Structural integrity of OP:SDS complexes. (a) Particle diameter distributions determined by dynamic light scattering for OP in the presence of 0, 800, 1000, 1500 or 2000 molar equivalents of SDS. (b) Raw correlation data for the same five samples (PDIs = 0.339, 0.386, 0.408, 0.385, 0.414 from 0-2000 equivalents of SDS, respectively). As there is negligible change in particle diameter up to 2000 equivalents, the increased electrophoretic mobility observed in panel (c) can be attributed to an increase in surface charge. (c) Duplicate of the experiment shown in Fig. 2b with an additional condition at high SDS concentration, Lanes 1-11: increments of 200 equivalents of SDS added to OP cages, Lane 12: fully denatured protein, which was obtained by treatment with 65 mM SDS, equating to 130,000 equivalents. These results suggest that beyond the cavity loading capacity of 800 molecules, additional SDS can further associate with the surface of the protein, probably filling the pores and exposing some of the negatively charged head groups. Gel visualized with Coomassie blue. (d) Zeta potential comparison of empty and SDS-filled OP cages. At basic pH, the OP cage has a negatively charged surface (ζ = -27.7 ± 7.1 mV), consistent with the native gel electrophoresis. Upon encapsulation of 800 molecules of SDS there is negligible change to the surface charge (ζ = -28.5 ± 6.4 mV), which is consistent with lumenal internalization. (e) Native agarose gel of OP:SDS (1:800) complexes assembled at concentrations below and at the critical micelle concentration of SDS (4-5 mM in PBS). The consistent band mobility shows that complex structure is defined by the ratio of OP:SDS rather than the absolute concentration of SDS. Gel visualized with Coomassie blue.

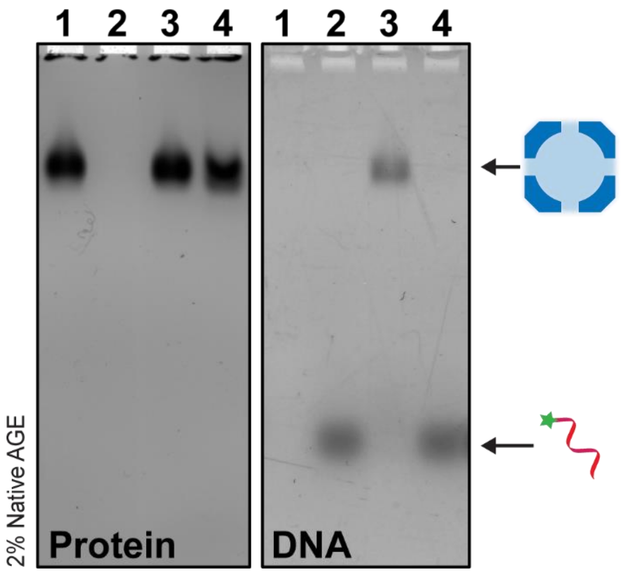

Supplementary Figure 2. Native agarose gel stained with Coomassie blue for protein (left) and visualized by Atto488 fluorescence for labelled ssDNA probe (right). Lane 1 – OP; Lane 2 – Atto488-labelled DNA; Lane 3 – DNA + OP; Lane 4 – DNA + pre-formed OP:SDS complexes. While empty OP cages internalize the DNA quantitatively, the SDS-filled cages cannot encapsulate the DNA probe.

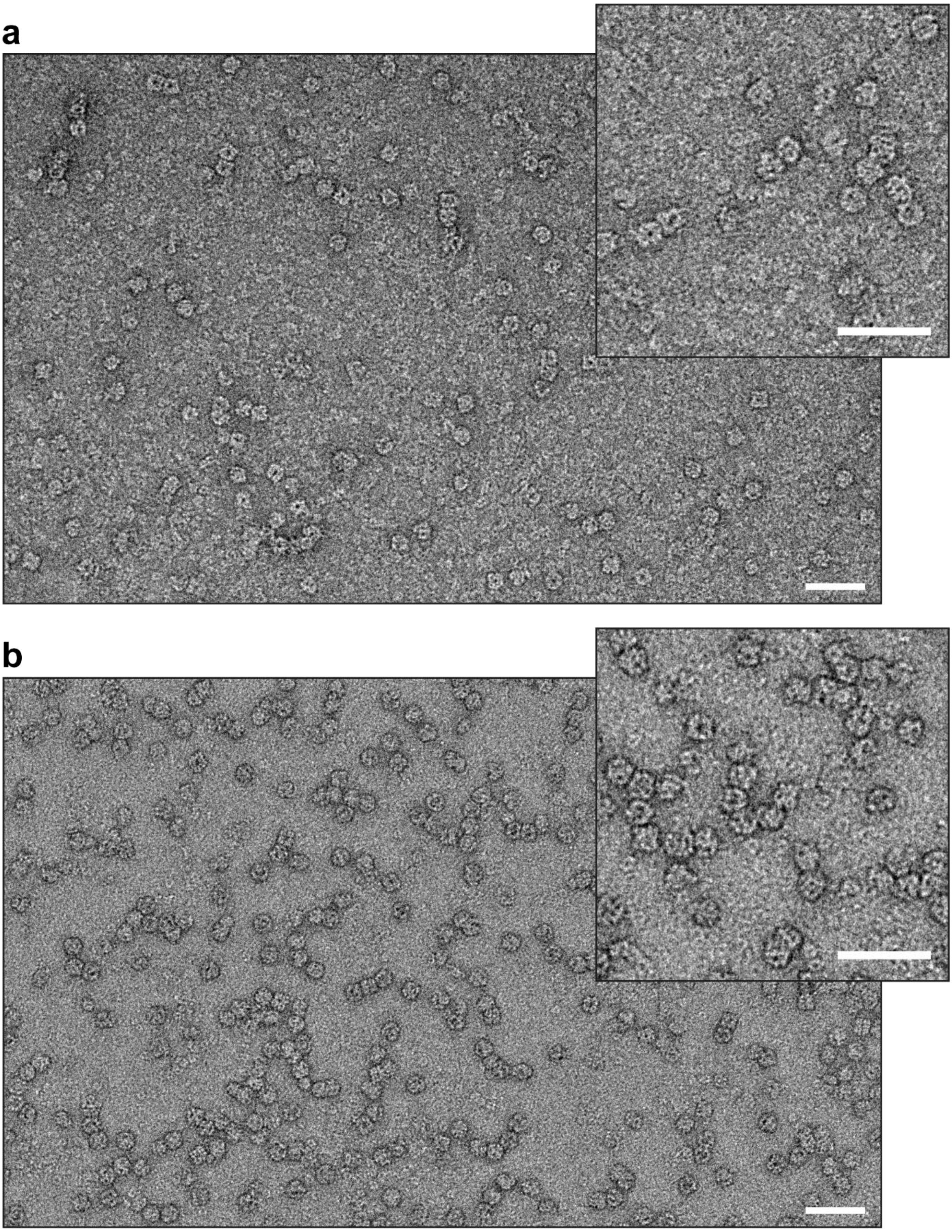

Supplementary Figure 3. Additional TEM images of OP and OP:SDS complexes. Negatively stained transmission electron micrographs of (A) OP and (B) OP:SDS. All scale bars are 50 nm.

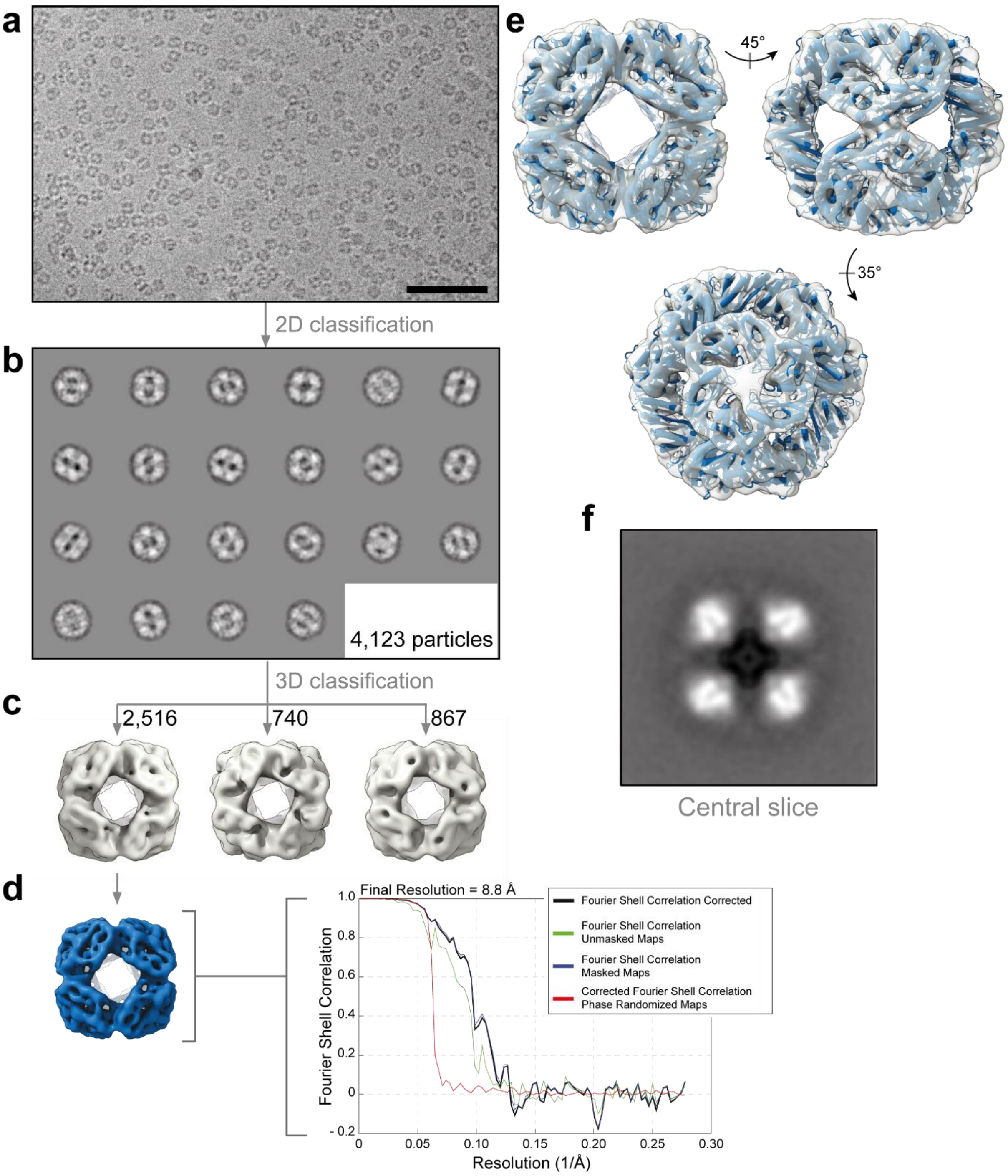

Supplementary Figure 4. Cryo-EM of empty OP capsids. (a) Representative cryo-electron micrograph of OP particles in vitreous ice, scale bar = 100 nm. (b) The best classes from three consecutive rounds of 2D classification (22 classes, 4,123 particles). (c) 3D classes with imposed octahedral symmetry. (d) The best 3D class was refined and postprocessed to a resolution of 8.8 Å, as determined by the gold-standard FSC 0.143 criterion. (e) The resulting 3D reconstruction (transparent surface) overlaps well with the reported crystal structure of OP (blue ribbons and cylinders, PDB: 6FDB). (f) Central slice through the refined map, showing the empty interior cavity.

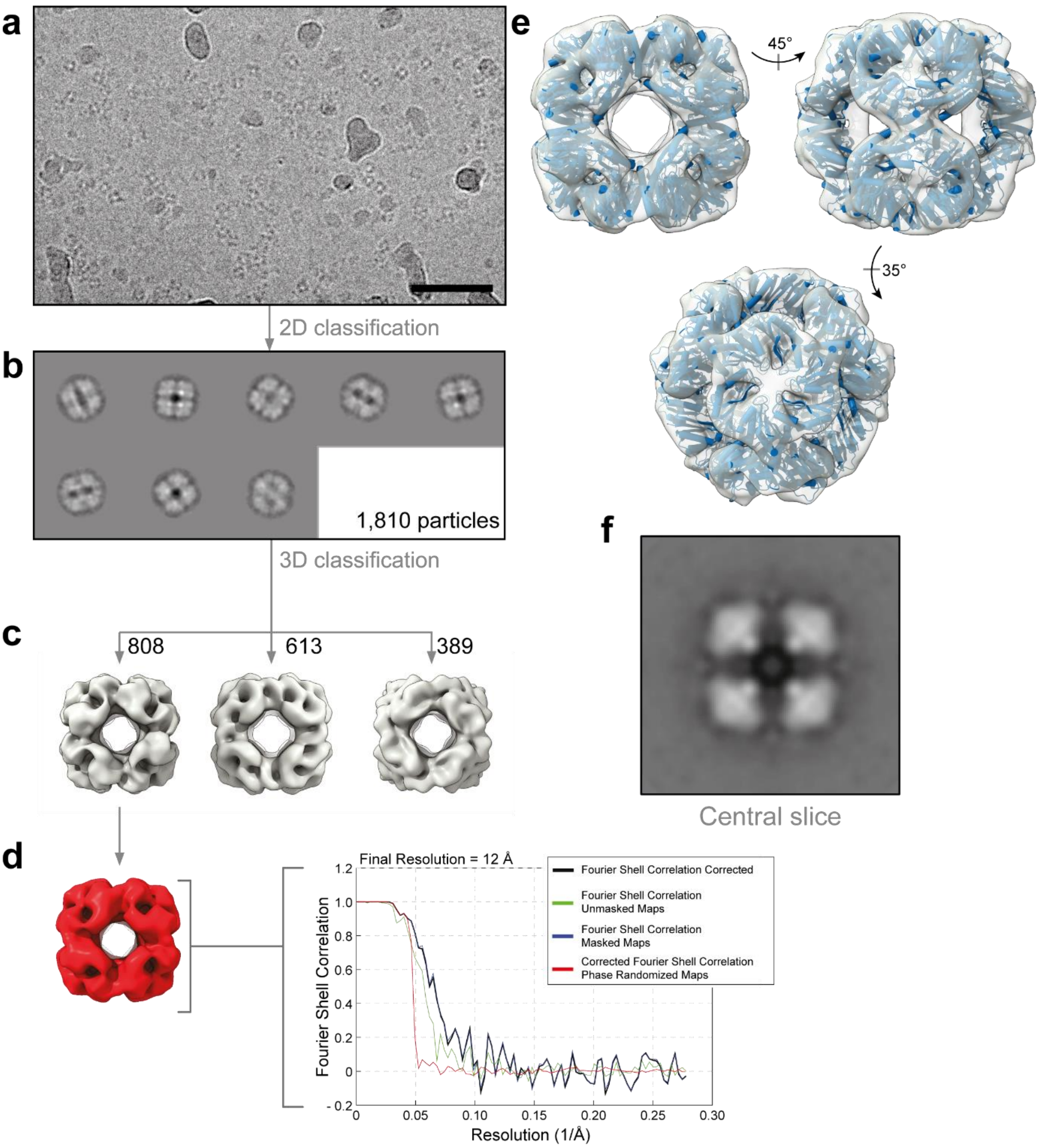

Supplementary Figure 5. Cryo-EM of OP:SDS complexes. (a) Representative cryo-electron micrograph of OP:SDS complexes in vitreous ice, scale bar = 100 nm. (b) The best classes from three consecutive rounds of 2D classification (8 classes, 1,810 particles). (c) 3D classes with imposed octahedral symmetry. (d) The best 3D class was refined and postprocessed to a resolution of 12 Å, as determined by the gold-standard FSC 0.143 criterion. (e) The resulting 3D reconstruction (transparent surface) overlaps well with the reported crystal structure of OP (blue ribbons and cylinders, PDB: 6FDB). (f) Central slice through the refined map. The lower resolution and data quality for this sample, which reflect challenges concentrating and freezing OP:SDS complexes, precludes reliable comparison with OP (Supplementary Fig. 4) or OP:SDS:CS (Supplementary Fig. 12). Nevertheless, multiple datasets were collected, all of which confirmed the structural integrity of the protein cage.

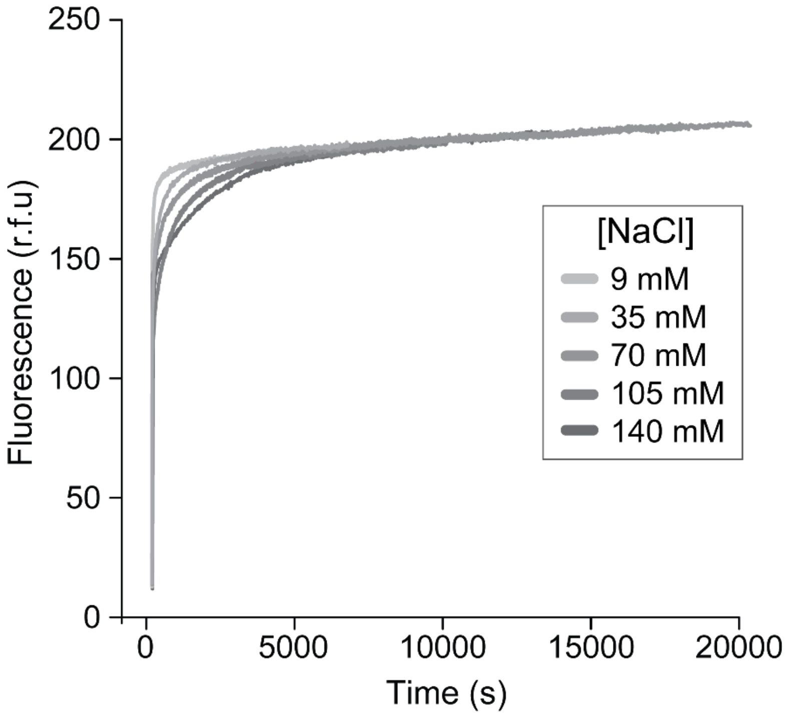

Supplementary Figure 6. Nile Red fluorescence monitored over time after addition of 800 equivalents of SDS to 240 nM OP in the presence of 9-140 mM NaCl. The Nile Red concentration is 960 nM. The sample was excited at 535 nm and emission was monitored at 620 nm. The formation of OP:SDS complexes is faster at lower ionic strength, as expected for electrostatically driven self-assembly.

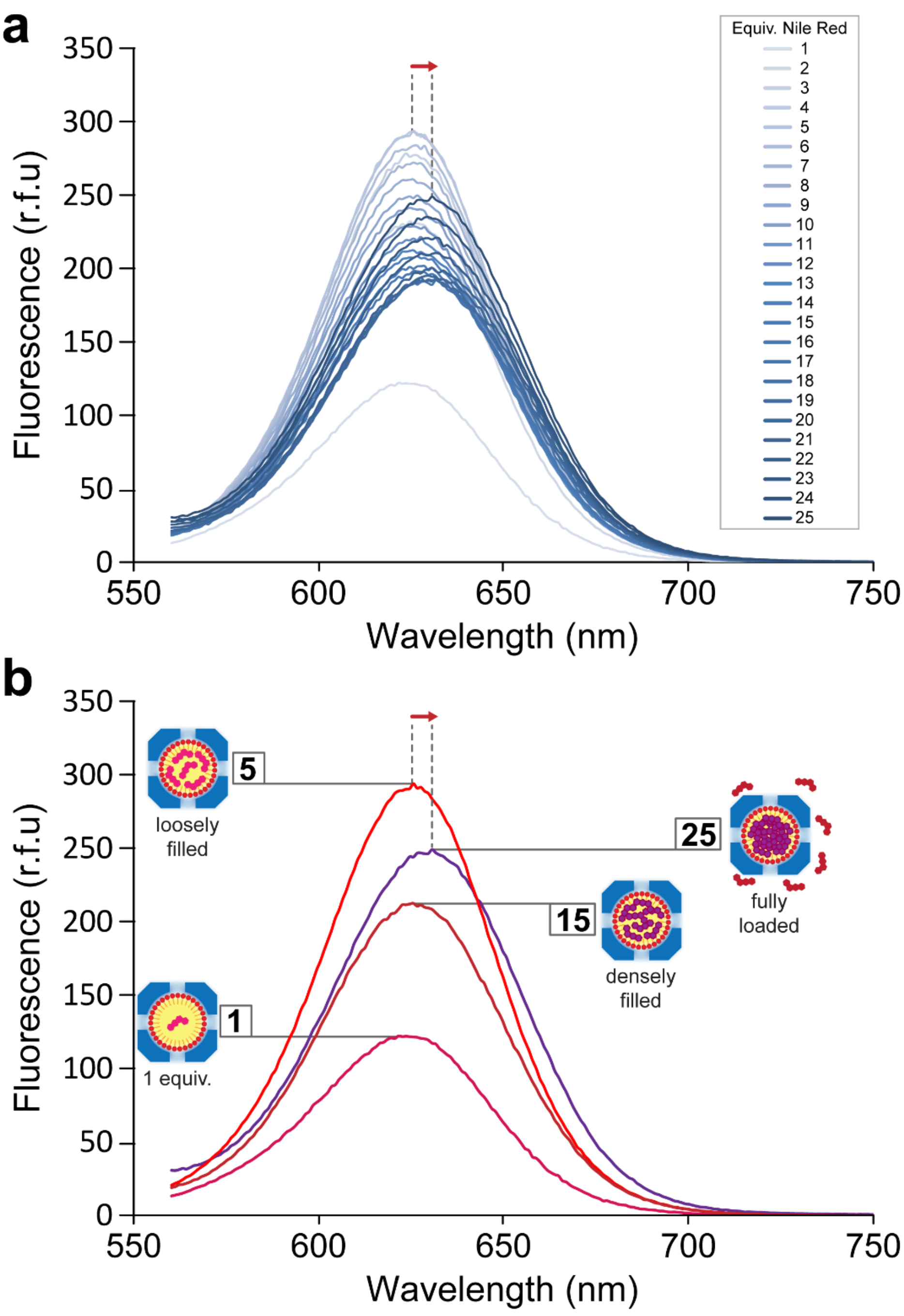

Supplementary Figure 7. Representative spectra from titration of OP:SDS with Nile Red. (a) Spectra obtained after addition of single equivalents, from 1 to 25, of Nile Red to pre-formed OP:SDS complexes at 240 nM capsid in PBS. The red arrow highlights a redshift in the emission maximum, which is observed above the loading capacity of the OP:SDS complex (*ca.* 20 equiv.), indicating that the additional molecules are present in bulk solution. (b) Four selected spectra show the distinct stages of the loading process indicated in Fig. 3d. The numbers 1, 5, 15 and 25 indicate the molar equivalents of Nile Red with respect to capsid.

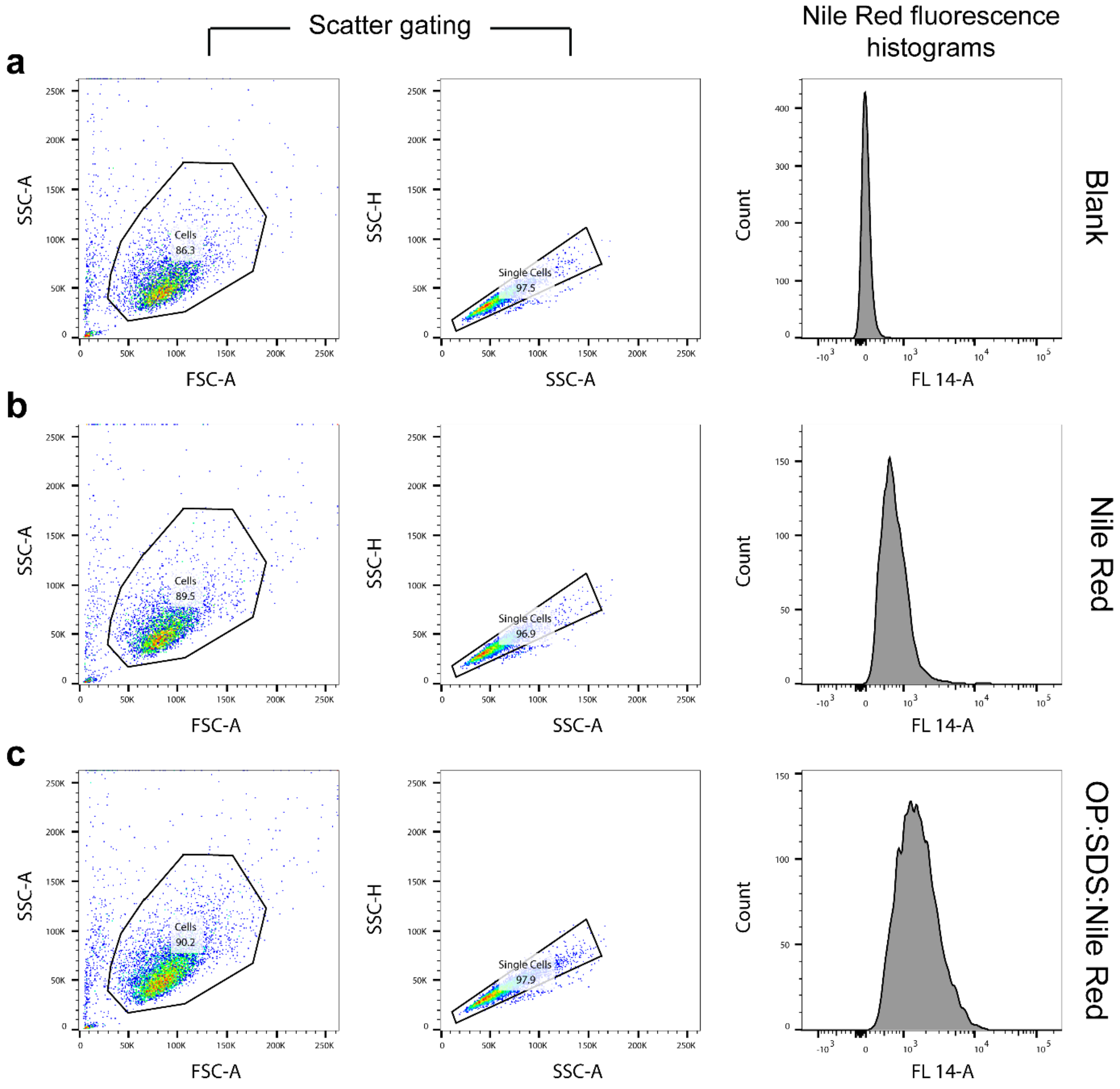

Supplementary Figure 8. Representative flow cytometry dataset. All sample gating and resulting histograms of Nile Red fluorescence for the treatment of HeLa cells with (a) PBS buffer (blank), (b) Nile Red (500 nM), and (c) OP:SDS:Nile Red (1:800:5, at 500 nM Nile Red). A minimum of 5,000 cells were analyzed for each sample replicate.

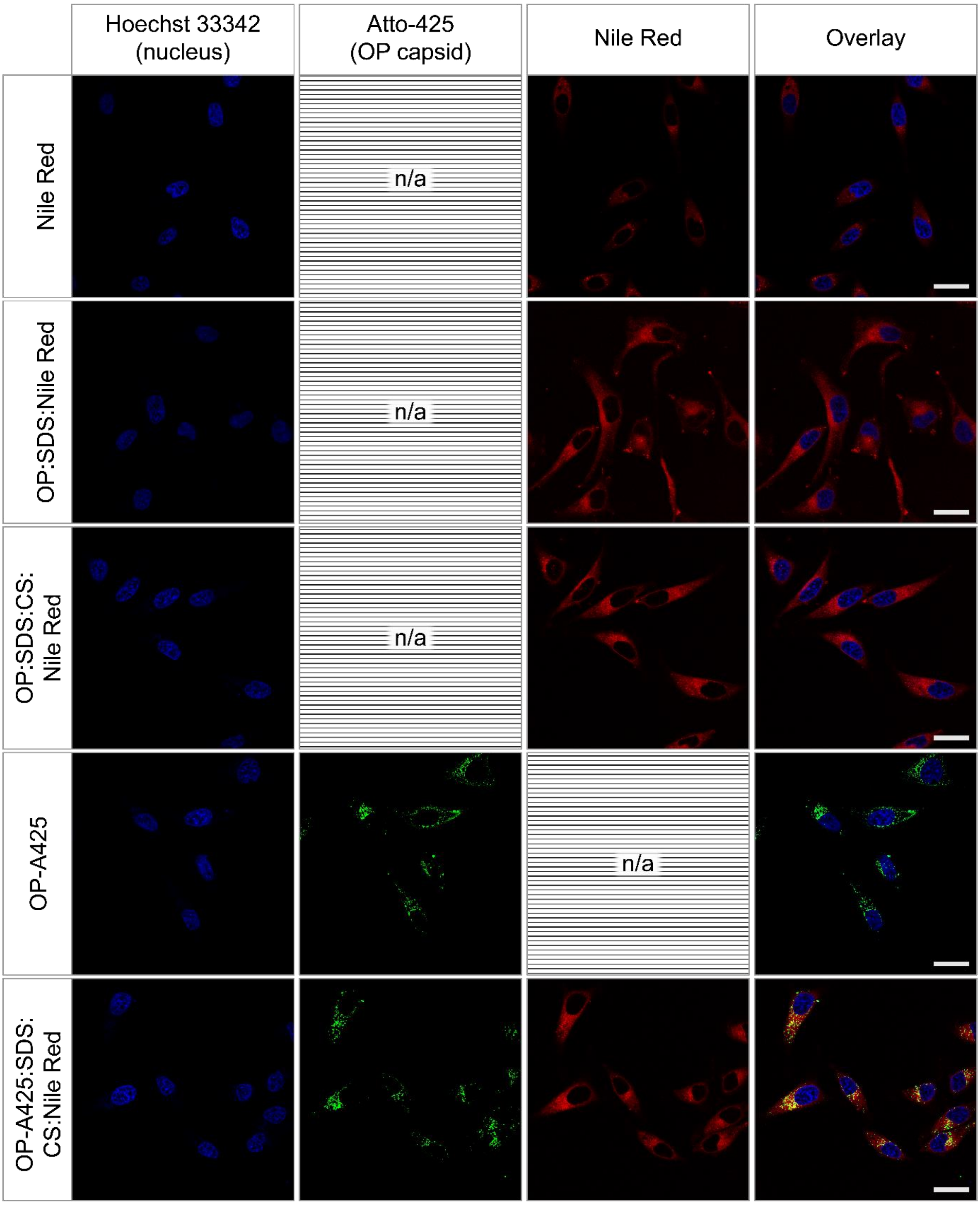

Supplementary Figure 9. Additional confocal fluorescence micrographs. In each case cells were exposed to 500 nM Nile Red. For the surfactant-filled OP cages the molar ratios are OP:SDS:Nile Red = 1:800:5, OP:SDS:CS:Nile Red = 1:600:200:5, and OP-A425:SDS:CS:Nile Red = 1:600:200:5. Atto425-labelled OP capsids have an average of 1.9 dyes per capsid. n/a refers to channels that are not applicable as the corresponding fluorophore was not present. All scale bars are 30 μm.

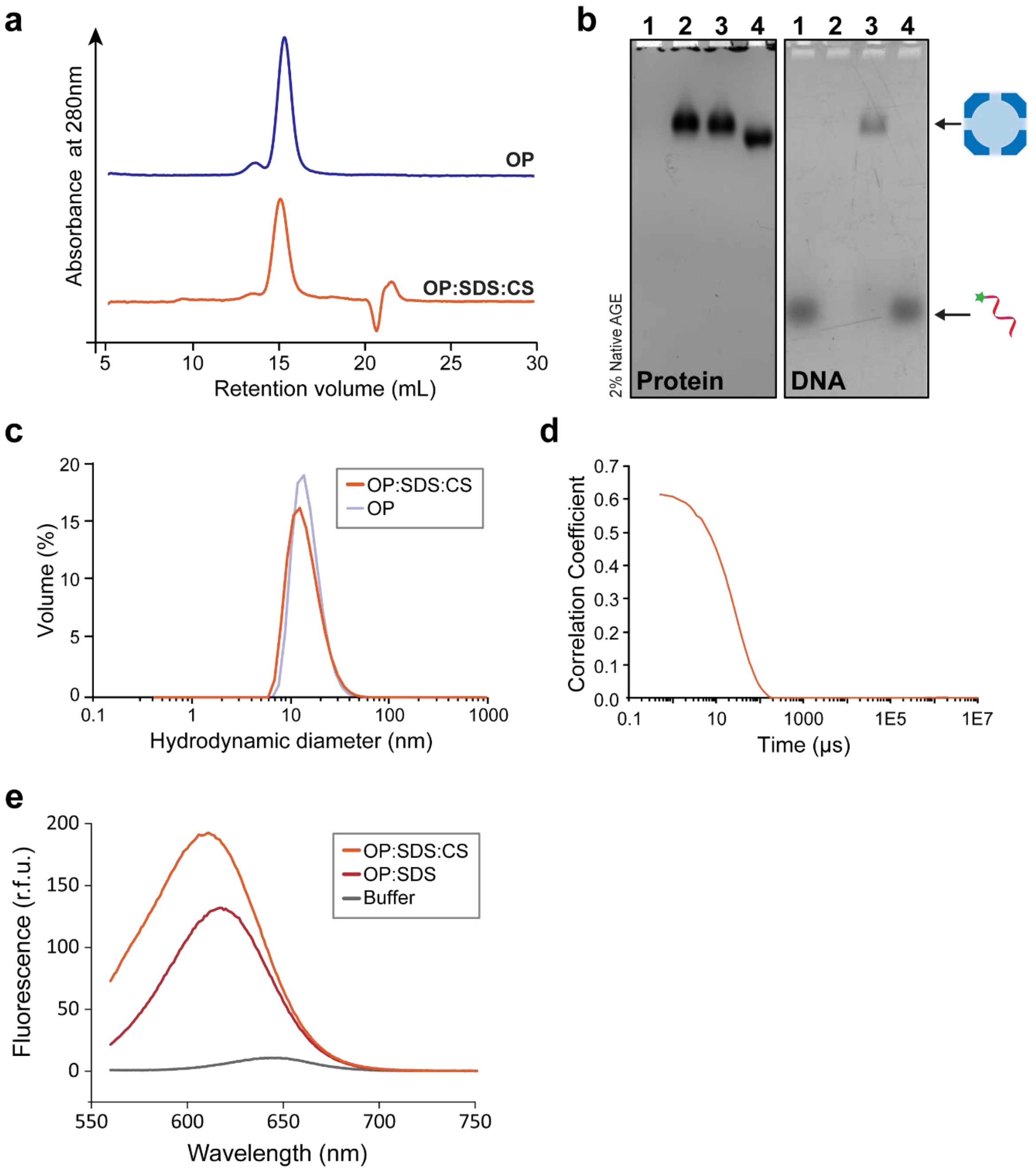

Supplementary Figure 10. Characterization of OP:SDS:CS complexes. (a) Size-exclusion chromatograms of OP and OP:SDS:CS complexes. The negative and positive peaks at 21 mL are due to the elution of DMSO, which is not present in the running buffer. (b) Native agarose gel stained with Coomassie blue for protein (left) and visualized by Atto488 fluorescence for labelled ssDNA probe (right). Lane 1 – Atto488-labelled DNA only; Lane 2 – OP; Lane 3 – DNA + OP, Lane 4 – DNA + pre-formed OP:SDS complexes. While empty OP cages can internalize the DNA quantitatively, the SDS:CS-filled cages cannot encapsulate the DNA probe. (c) Dynamic light scattering diameter distribution for OP:SDS:CS complexes (mean diameter = 14.0 nm; PDI = 0.237), with OP shown in light blue for reference (mean diameter = 14.3; PDI = 0.339), the average. (d) Raw correlation data for OP:SDS:CS complexes. (e) Fluorescence spectra of Nile Red in buffer (gray) and in the presence of OP:SDS cages (red) and OP:SDS:CS cages (orange). As the Nile Red is encapsulated within the complexes, the increase in fluorescence and blueshift of the Nile Red emission maximum indicate that CS is incorporated into the protein-scaffolded micelle.

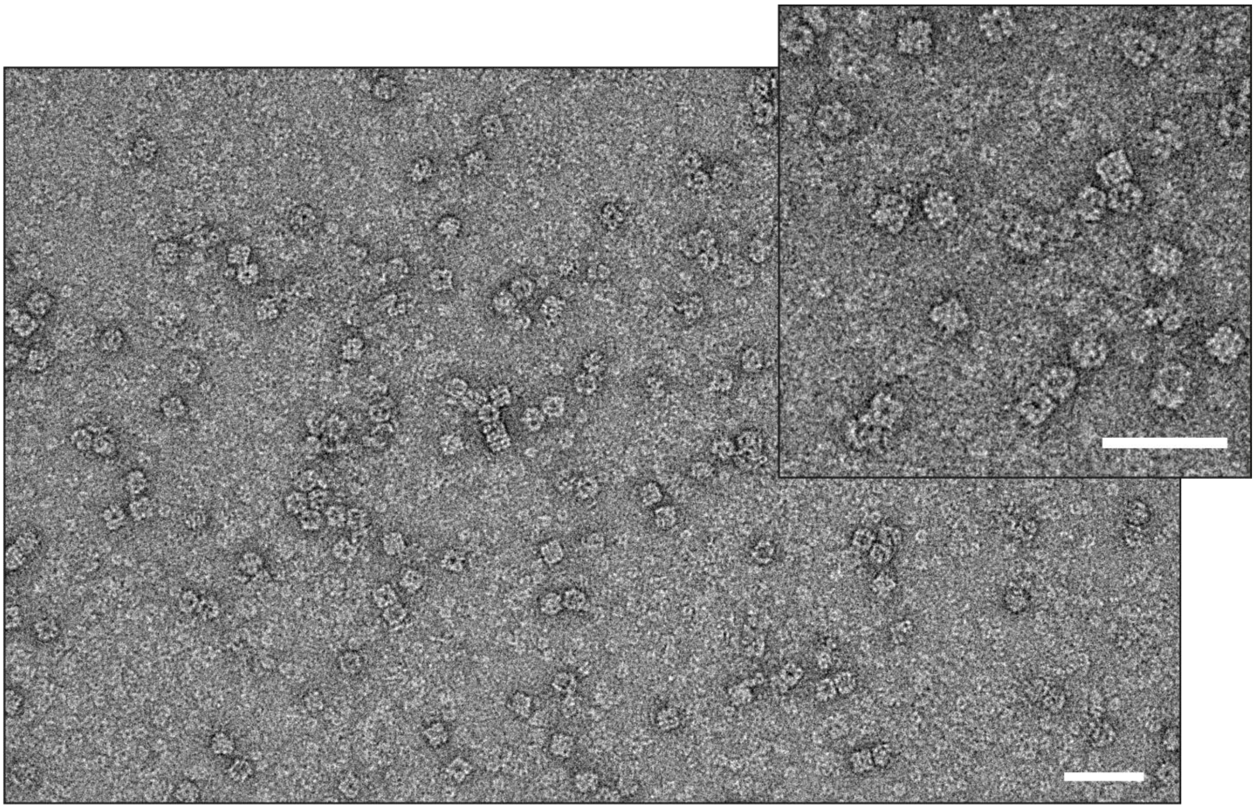

Supplementary Figure 11. Negatively stained transmission electron micrographs of OP:SDS:CS complexes. Both scale bars are 50 nm.

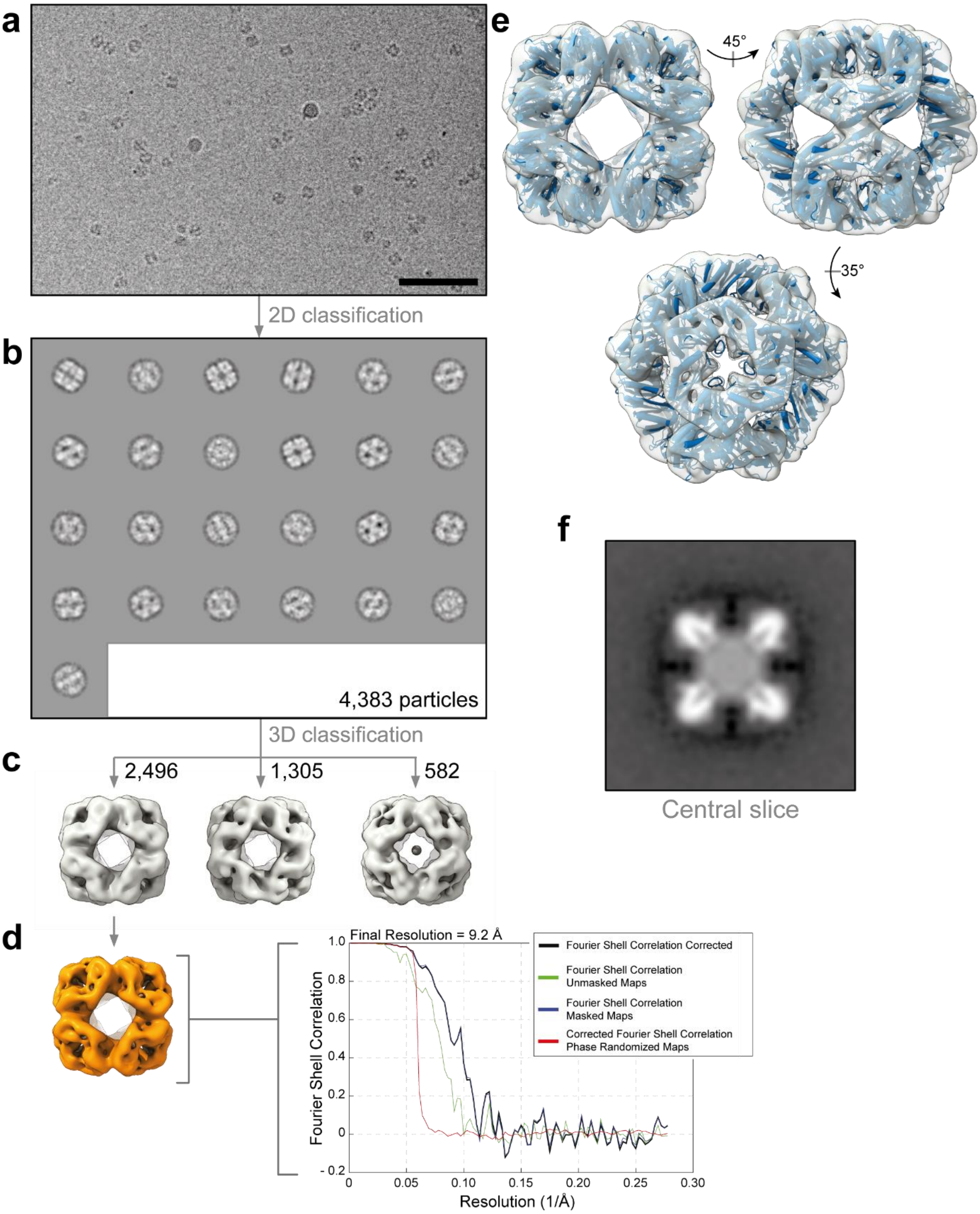

Supplementary Figure 12. Cryo-EM of OP:SDS:CS complexes. (a) Representative cryo-electron micrograph of OP:SDS:CS complexes in vitreous ice, scale bar = 100 nm. (b) The best classes from three consecutive rounds of 2D classification (25 classes, 4,383 particles). (c) 3D classes with imposed octahedral symmetry. (d) The best 3D class was refined and postprocessed to a resolution of 9.2 Å, as determined by the gold-standard FSC 0.143 criterion. (e) The resulting 3D reconstruction (transparent surface) overlaps well with the reported crystal structure of OP (blue ribbons and cylinders, PDB: 6FDB). (f) Central slice through the refined map, which shows a clear increase in lumenal density compared to the empty OP cage (Supplementary Fig. 4), consistent with internalization of the surfactants.

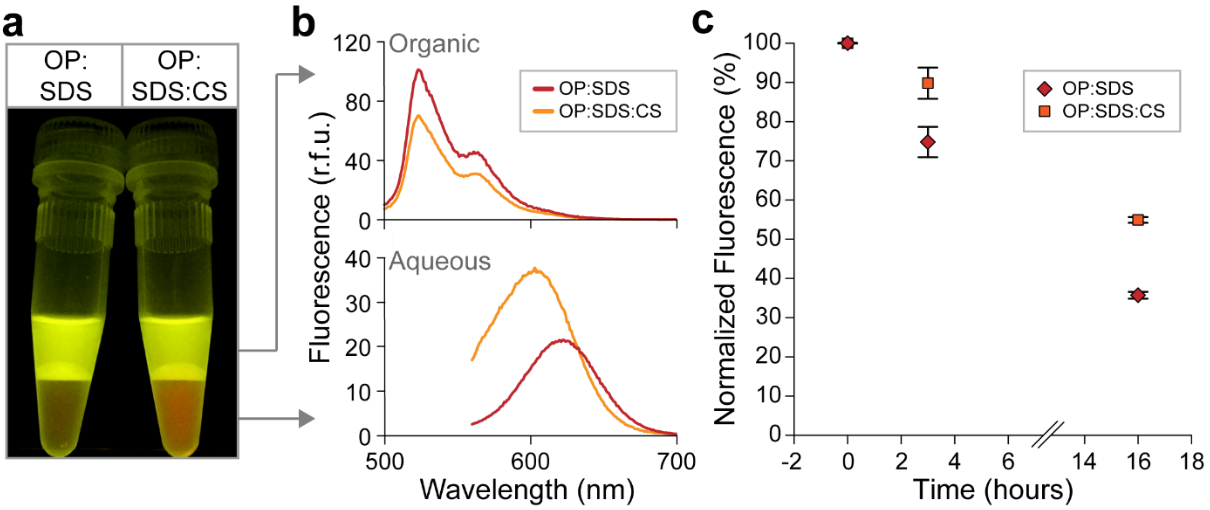

Supplementary Figure 13. Cargo release from OP:SDS and OP:SDS:CS complexes. (a) Photograph of OP:SDS:NR and OP:SDS:CS:NR samples incubated with hexane overnight, imaged by UV transillumination. Yellow Nile Red emission is observed in the upper organic phase, while OP-encapsulated Nile Red gives its typical red fluorescence in the lower aqueous phase. (b) Emission spectra for the organic and aqueous phases from both samples, obtained with excitation wavelengths of 470 and 535 nm respectively. (c) Nile Red fluorescence in the aqueous phase as a function of time, shown as percentage of the fluorescence of the same sample without incubation with hexane. The addition of cholesterol sulfate reduces the off-rate for Nile Red encapsulation, resulting in retention of 20% more Nile Red after 16 hours (n=3, error bars: standard deviation).

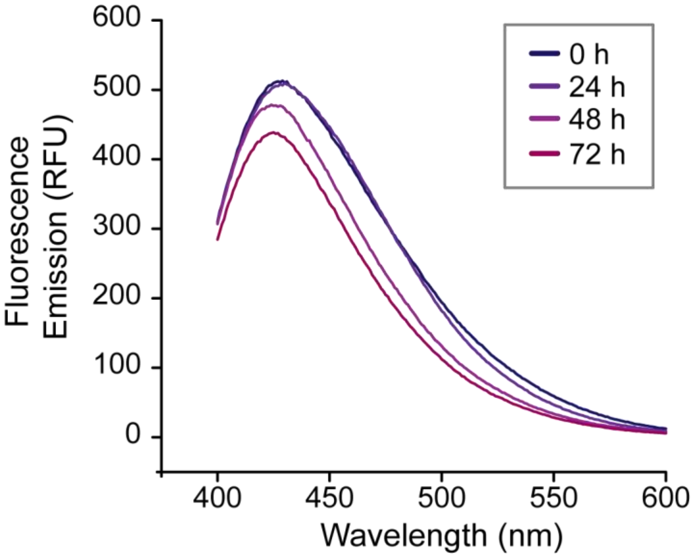

Supplementary Figure 14. Fluorescence spectra of lapatinib-loaded OP:SDS:CS complexes that have been dialyzed against bovine serum albumin (BSA) containing medium for 24, 48 or 72 hours compared to the undialyzed control.

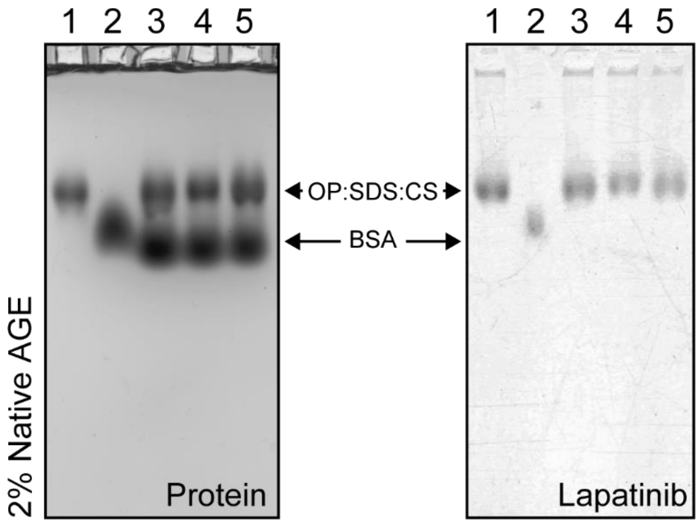

Supplementary Figure 15. Native agarose gel of OP:SDS:CS complexes, lapatinib and BSA. Lane 1 – OP:SDS:CS:lapatinib (1:600:200:10); Lane 2 – BSA:lapatinib (1:1); Lane 3 – [OP:SDS:CS:lapatinib] + BSA; Lane 4 – [OP:SDS:CS + BSA] + lapatinib; Lane 5 – [BSA:lapatinib] + OP:SDS:CS. In lanes 3-5, the species grouped in square brackets were incubated together for 1 hour before addition of another molecule, which was then incubated for a further hour before gel analysis. These data reveal that in all cases lapatinib is preferentially encapsulated by OP:SDS:CS complexes rather than binding to BSA, corroborating the results from the fluorescence experiment shown in Supplementary Fig. 14.

Supplementary Table 1

Macromolecular structural data from cryo-electron microscopy have been deposited in the EMDB with the accession codes: OP, EMD-10723; OP:SDS, EMD-10724; OP:SDS:CS, EMD-10725.

Data collection, refinement and validation statistics

	OP (EMDB-10723)	OP:SDS (EMDB-10724)	OP:SDS:CS (EMDB-10725)
**Data collection and processing**			
Magnification	62’000x	62’000x	62’000x
Voltage (kV)	200	200	200
Electron exposure (e–/Å^2)	30	35	30
Defocus range (μm)	-1.8 to -3.3	-1.8 to -3.3	-1.8 to -3.3
Pixel size (Å)	1.8	1.8	1.8
Symmetry imposed	O	O	O
Initial particle images (no.)	45’328	14’082	12’174
Final particle images (no.)	2’516	808	2’499
Map resolution (Å)	8.8	11.6	9.2
FSC threshold	0.143	0.143	0.143
Map resolution range (Å)	∞ - 8.8	∞ - 11.6	∞ - 9.2

PDB model 6FDB was used for rigid body fitting.
