# Supplementary material for: Two-tier supramolecular encapsulation of small molecules in a protein cage
Source: Nat Commun. 2020 Oct 26;11:5410. doi: 10.1038/s41467-020-19112-1 (PMC7588467; doi:10.1038/s41467-020-19112-1)
Supplement: Supplementary file 3 — Reporting Summary [file 41467_2020_19112_MOESM3_ESM.pdf]

nature research

Corresponding author(s): Donald Hilvert

Last updated by author(s): Sep 3, 2020

Reporting Summary

Nature Research wishes to improve the reproducibility of the work that we publish. This form provides structure for consistency and transparency in reporting. For further information on Nature Research policies, see Authors & Referees and the Editorial Policy Checklist .

Statistics

For all statistical analyses, confirm that the following items are present in the figure legend, table legend, main text, or Methods section.

n/a	Confirmed	
☐	☒	The exact sample size (*n*) for each experimental group/condition, given as a discrete number and unit of measurement
☐	☒	A statement on whether measurements were taken from distinct samples or whether the same sample was measured repeatedly
☒	☐	The statistical test(s) used AND whether they are one- or two-sided<br>*Only common tests should be described solely by name; describe more complex techniques in the Methods section.*
☒	☐	A description of all covariates tested
☒	☐	A description of any assumptions or corrections, such as tests of normality and adjustment for multiple comparisons
☐	☒	A full description of the statistical parameters including central tendency (e.g. means) or other basic estimates (e.g. regression coefficient) AND variation (e.g. standard deviation) or associated estimates of uncertainty (e.g. confidence intervals)
☒	☐	For null hypothesis testing, the test statistic (e.g. *F*, *t*, *r*) with confidence intervals, effect sizes, degrees of freedom and *P* value noted<br>*Give P values as exact values whenever suitable.*
☒	☐	For Bayesian analysis, information on the choice of priors and Markov chain Monte Carlo settings
☒	☐	For hierarchical and complex designs, identification of the appropriate level for tests and full reporting of outcomes
☒	☐	Estimates of effect sizes (e.g. Cohen's *d*, Pearson's *r*), indicating how they were calculated

Our web collection on statistics for biologists contains articles on many of the points above.

Software and code

Policy information about availability of computer code

Data collection	DSLR Remote Pro v1.4 (Fluorescence Gel images), Epson SilverFast v8.0.1r16 (Coomassie Gel images), Biorad ChromLab v4.0.0.25 Std Edition (Size-exclusion Chromatography), FEI EPU Software v1.12 (Electron microscopy), Felix X32 PTI software v1.2 Build 56 (Fluorimetry), BD FACSDiva 8.0.1 (Flow cytometry), Leica Application Suite X v3.7.0.20979 (Confocal Microscopy), Molecular Devices SoftMax Pro v5.4 (Microplate reader)
Data analysis	Leica Application Suite X v3.7.0.20979 (Confocal Microscopy), Adobe Photoshop 19.1.5 (Gel images), FlowJo v10.1 (Flow cytometry data), Microsoft Excel - MS Office Professional Plus 2013 (Fluorimetry, flow cytometry, size-exclusion chromatography, cytotoxicity), OriginPro b9.3.1.273 (Fluorimetry, flow cytometry, cytotoxicity), UCSF Chimera v1.10.2 Build 40686 (Protein structure), UCSF ChimeraX v0.91 (Protein structure), Relion v3.0 (cryo-electron microscopy), Fiji 1.51n (Image processing), Phenix v1.13-2998 (cryo-electron microscopy)

For manuscripts utilizing custom algorithms or software that are central to the research but not yet described in published literature, software must be made available to editors/reviewers. We strongly encourage code deposition in a community repository (e.g. GitHub). See the Nature Research guidelines for submitting code & software for further information.

Data

Policy information about availability of data

All manuscripts must include a data availability statement. This statement should provide the following information, where applicable:

- Accession codes, unique identifiers, or web links for publicly available datasets
- A list of figures that have associated raw data
- A description of any restrictions on data availability

The data supporting the conclusions of this work can be found in the Figures and Supplementary Information. Additional data are available from the corresponding author upon request. Macromolecular structural data from cryogenic electron microscopy have been deposited in the EMDB with the accession codes: OP, EMD-10723; OP:SDS, EMD-10724; OP:SDS:CS, EMD-10725. The previously reported OP crystal structure is available from the PDB, accession code: 6FDB.

Field-specific reporting

Please select the one below that is the best fit for your research. If you are not sure, read the appropriate sections before making your selection.

- [x] Life sciences
- [ ] Behavioural & social sciences
- [ ] Ecological, evolutionary & environmental sciences

For a reference copy of the document with all sections, see nature.com/documents/nr-reporting-summary-flat.pdf

Life sciences study design

All studies must disclose on these points even when the disclosure is negative.

Sample size	No sample-size calculations were used. Enough replicate measurements or samples were analyzed to determine that the data were sufficiently reproducible.
Data exclusions	With the exception of flow cytometry gating (which is described in detail below), no data were excluded from analysis.
Replication	Experimental findings were reproduced on multiple occasions using both technical and biological replicates, all data were reproduced successfully. The number of replicates varied for each experiment with a minimum of three replicates.
Randomization	For all 'life science' experiments in this study, namely flow cytometry, confocal microscopy and cell viability assays, sample positions on 96- and 24-well culture plates were randomized to avoid any edge effects.
Blinding	The investigators were not blinded during data analysis. Blinding was not necessary, as the data collected were not affected by knowledge of sample identity at the time of measurement nor the results by knowledge of sample identity at the time of analysis.

Reporting for specific materials, systems and methods

We require information from authors about some types of materials, experimental systems and methods used in many studies. Here, indicate whether each material, system or method listed is relevant to your study. If you are not sure if a list item applies to your research, read the appropriate section before selecting a response.

Materials & experimental systems

n/a	Involved in the study	
x		Antibodies
	x	Eukaryotic cell lines
x		Palaeontology
x		Animals and other organisms
x		Human research participants
x		Clinical data

Methods

n/a	Involved in the study	
x		ChIP-seq
	x	Flow cytometry
x		MRI-based neuroimaging

Eukaryotic cell lines

Policy information about cell lines

Cell line source(s)	HeLa cells were purchased from ATCC
Authentication	Cells were not authenticated after purchase
Mycoplasma contamination	None observed. Cells were monitored periodically for mycoplasma contamination by growth rate and viability.
Commonly misidentified lines (See ICLAC register)	None were used

Flow Cytometry

Plots

Confirm that:

- [x] The axis labels state the marker and fluorochrome used (e.g. CD4-FITC).
- [x] The axis scales are clearly visible. Include numbers along axes only for bottom left plot of group (a 'group' is an analysis of identical markers).
- [x] All plots are contour plots with outliers or pseudocolor plots.
- [x] A numerical value for number of cells or percentage (with statistics) is provided.

Methodology

Sample preparation	After incubation with samples, cells were washed with PBS and trypsinized (0.05% Trypsin-EDTA (Thermo Fisher Scientific, USA), 4 minutes at 37 °C). Cells were collected in cold culture medium and washed twice with cold PBS before resuspension in flow cytometry buffer (PBS with 5% FBS).
Instrument	BD LSR Fortessa
Software	Data was collected using the BD FACSDiva Software and analyzed using FlowJo Version 10.1
Cell population abundance	Cells were not sorted. They were only analyzed for median fluorescence intensity.
Gating strategy	Scatter gating was used to remove any dead cells or other particulates (SSC-A/FSC-A), and to remove cell doublets (SSC-H/SSC-A). The exact gate values depended on the instrument calibration and settings. An exemplary dataset with gating is shown in the Extended Data section (Extended Data Figure 5).

- [x] Tick this box to confirm that a figure exemplifying the gating strategy is provided in the Supplementary Information.
